# 562. Invasive Fungal Infection Increases Civilian Mortality Risk After Trauma and Burn Injury

**DOI:** 10.1093/jbcr/irag033.302

**Published:** 2026-04-06

**Authors:** Allison Frederick, Steven A Kahn, Rohit Mittal

**Affiliations:** Medical University of South Carolina, Charleston, SC; Medical University of South Carolina, Charleston, SC; South Carolina Burn Center at Medical University of South Carolina, Charleston, SC

## Abstract

**Introduction:**

Invasive fungal infections (IFI) are increasingly diagnosed in trauma and burn patients with serious, often deadly prognosis. Most knowledge comes from military data or small case series. This study captures a generalizable view of civilian IFI after trauma and burn injury using a multi-institutional database to evaluate incidence, mortality and associated injuries.

**Methods:**

Using TriNetX database, we identified patients with traumatic and burn injury (traumatic amputations, open fractures and burns) from 2002-2025. IFI was defined as fungal mycosis diagnosis with systemic antifungal treatment, compared to controls without mycoses or antifungal treatment. Cohorts were propensity score matched (1:1) for demographics and 1-year in-hospital mortality was compared to determine risk ratios, 95% confidence intervals and significance. Subgroup analyses examined injury type, mechanism and fungal infection type.

**Results:**

The traumatic injury cohort contained 97 384 patients; controls contained 9 297 567, with IFI incidence of 1.05%. For all traumatic and burn injuries, 1-year in-hospital mortality with IFI was 3.13% versus 1.08% in controls (RR 2.91, *p*<.0001). When stratified by injury type with IFI, significantly higher mortality risk was found for burn injuries (RR 7.96, incidence 0.46%, *p*<.0001), traumatic amputations (RR 3.32, incidence 1.91%, *p*<.0001) and traumatic open fractures (RR 1.62, incidence 0.95%, *p*=.01) compared to respective cohorts without IFI.

**Conclusions:**

In this two-decade civilian study, overall IFI incidence after traumatic injury is 1% with three-fold increased mortality. Burn injury shows eight-fold mortality increase with IFI, traumatic amputations three-fold, and open fractures less than two-fold. Given these significant mortality rates, clinicians must maintain high suspicion for IFI in traumatic injuries, warranting further investigation to determine which patients would benefit from prophylaxis and aggressive treatment.

**Applicability of Research to Practice:**

This study provides multi-institutional evidence for IFI risk stratification in civilian trauma, identifying injury types requiring heightened surveillance and intervention strategies.

**Funding for the study:**

N/A.

